# The NLRP3 Inflammasome Transmits Sterile Inflammation Signals to Sustain Proper Mitochondrial Electron Transport Chain Function and Influences Cellular Metabolism

**DOI:** 10.1007/s12015-025-10948-y

**Published:** 2025-07-28

**Authors:** Adrian Konopko, Michalina Kazek, Emilia Waraksa-Zasada, Agnieszka Łukomska, Janina Ratajczak, Magdalena Kucia, Mariusz Z. Ratajczak

**Affiliations:** 1 Center for Preclinical Studies and Technology, Laboratory of Regenerative Medicine, Medical University of Warsaw, Warsaw, Poland; 2 Stem Cell Institute at James Graham Brown Cancer Center, University of Louisville, 500 S. Floyd Street, Rm. 107, Louisville, KY 40202 USA

**Keywords:** Nlrp3 inflammasome, Oxygen consumption rate, Electron transport chain complex, Mitochondria, Hematopoiesis, Glycolysis

## Abstract

The Nlrp3 inflammasome is a pattern recognition receptor (PRR) and an important component of innate immunity, located in the cytoplasm of various cell types, including those derived from hematopoietic lineages. It is highly expressed in hematopoietic stem/progenitor cells (HSPCs) and in the cells that constitute the bone marrow (BM) hematopoietic microenvironment. The Nlrp3 inflammasome plays a role in several biological processes depending on the level of activation. At low activation levels, within the so-called “hormetic beneficial zone,” it positively regulates the trafficking of HSPCs, as seen during mobilization, homing, and engraftment following transplantation. It is also essential for maintaining a pool of HSPCs in the bone marrow (BM), and we have reported that Nlrp3 knockout (KO) mice demonstrate a decrease in the number of these cells. The primary mediators activated and released from the Nlrp3 inflammasome are interleukin-1 beta (IL-1β) and interleukin-18 (IL-18). Additionally, we have suggested that by promoting gasdermin channels in the cell membrane, multiple factors are released, including danger-associated molecular pattern molecules (DAMPs) or alarmins, which activate the corresponding receptors expressed on cell membranes. This process of creating “alarmin fog” in the hematopoietic microenvironment is responsible for the biological effects of this PRR through positive feedback that activates cell surface receptors, elevating intracellular ROS signaling. Here, we report for the first time that Nlrp3 inflammasome knockout mice exhibit defects in oxidative consumption rates. This was paralleled by a decreased level of expression of the mitochondria-encoded cytochrome b gene (CYTB), which encodes a crucial component of complex III in the electron transport chain (ETC). This data highlights the role of the Nlrp3 inflammasome in regulating cell metabolism by ensuring the proper “tonic activation” of the electron transport chain in mitochondria.

**Graphical Abstract:**

A graphical abstract has been prepared by using the application BioRender.

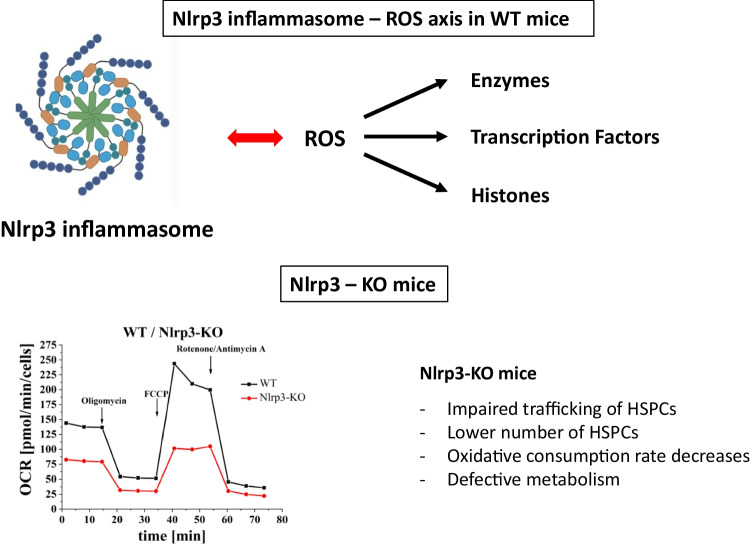

## Introduction

Inflammasomes are a family of multiprotein complexes that are part of the innate immune system's pattern recognition receptors (PRRs), which identify microbe-related pathogen-associated molecular patterns (PAMPs) or danger-associated molecular patterns (DAMPs) originating from damaged host cells [1–4]. They reside in the cytoplasm, and their activation triggers the activation of caspase-1 and the release of mature forms of interleukin-1 beta (IL-1β) and interleukin-18 (IL-18). Furthermore, caspase-1 cleaves the cell membrane pore-forming molecule gasdermin D, which is involved in releasing various factors from cells, including DAMPs or alarmin [1–4]. Depending on the level of activation, the Nlrp3 inflammasome can have beneficial effects on cell biology within the so-called hormetic zone, assisting in cell activation, migration, and metabolism [5–8]. Hormesis is a characteristic of many biological processes when there is exposure to increasing amounts of challenging or potentially harmful stimuli. The effects are biphasic: while a low dose of a challenging agent can be beneficial to cells, a high dose can be damaging. Interestingly, there is generally a favorable biological response to low exposures to a potential stressor—in what is called the “hormetic zone”—and several beneficial effects of innate immunity on hematopoiesis can be explained by this phenomenon [7, 8]. However, with increased activation levels, it may lead to cell damage through a pro-inflammatory form of apoptosis known as pyroptosis [1].

Several members of the inflammasome family have been identified, including NLRP1, AIM2, NLRC4, and the Nlrp3 inflammasome (Nlrp3) expressed in HSPCs [1, 3, 4]. Recently, we confirmed the expression of the Nlrp3 inflammasome using single-cell SeqRNA in highly purified hematopoietic stem cells [9]. Nlrp3 belongs to a group of receptors known as nucleotide-binding oligomerization domain-like receptors, or NOD-like receptors (NLRs), which are located in the cytoplasm in an inactive state. Upon activation, it forms a functional multiprotein complex composed of several Nlrp3 complex proteins (speck complexes) that include Nlrp3, ASC, and procaspase-1 [1, 3, 4]. The basic expression (priming) of the Nlrp3 inflammasome in some cells is regulated by the lipopolysaccharide-Toll-like receptor 4 (TLR4) axis [1]. The Nlrp3 inflammasome appears to be activated by changes in intracellular potassium levels resulting from potassium efflux through mechanosensitive ion channels, as observed in activated C5aR1 or purinergic P2X receptors located in the cell membrane [10]. Additionally, Nlrp3 is regulated by reactive oxygen species (ROS) produced during respiration in a NADPH oxidase-2 (NOX-2)-dependent manner expressed in mitochondria or associated with cell surface membrane receptors [11–13]. We envision that the Nlrp3 inflammasome could be involved in transferring sterile inflammation signals mediated by the activation of circulating and cell-expressed complement and purinergic signaling to regulate the metabolism of HSPCs.

It is reported that the Nlrp3 inflammasome, when activated at low levels within the so-called “hormetic beneficial zone,"positively regulates the trafficking of hematopoietic stem and progenitor cells, as observed during mobilization, homing, and engraftment following transplantation [14]. It is also crucial for maintaining a pool of HSPCs in the bone marrow (BM), and our research indicates that Nlrp3 knockout (KO) mice show a reduced number of these cells [14]. Another group has confirmed that the Nlrp3 inflammasome is vital for sustaining a pool of stem cells in zebrafish embryos and enhances the production of HSPCs in human hemogenic cultures [15]. This effect is linked to the release of IL-1β, which promotes hematopoiesis by engaging the hemogenic endothelium [15].

The primary mediators activated and released from the Nlrp3 inflammasome are interleukin-1 beta (IL-1β) and interleukin-18 (IL-18). We have also proposed that the formation of gasdermin channels in the cell membrane plays a crucial role in Nlrp3 inflammasome signaling, leading to the release of various factors from the cells, including DAMPs or alarmin [16]. These molecules activate corresponding receptors on the cell membrane that are responsible for the biological effects of this pattern recognition receptor (PRR) through positive feedback. Nevertheless, the exact mechanism by which the Nlrp3 inflammasome, as an intracellular PRR associated with caspase-1, regulates HSPCs beyond the role of IL-1β remains unclear. We postulate that the release of alarmin and cytokines through gasdermin pores may significantly modulate cell metabolism by creating an “alarmin fog” in the hematopoietic microenvironment, maintaining the “tonic activation” of e.g., mitochondria [16].

To better understand the role of the Nlrp3 inflammasome in regulating cell metabolism, which is crucial for providing the energy needed for cell proliferation, we examined the oxidative consumption rate (OCR) and mitochondrial function in Lin − bone marrow mononuclear cells (BMNCs). We report, for the first time, that Nlrp3 inflammasome knockout mice exhibit defects in OCR caused by decreased expression of cytochrome b protein, a key component of complex III in the electron transport chain (ETC). This data offers new insights into the role of the Nlrp3 inflammasome in the"tonic activation” of mitochondrial electron transport.

## Material and Methods

### Animals

Pathogen-free, 6–8-week-old female C57BL/6 J wild-type (WT) and Nlrp3-KO (B6.129S6-*Nlrp3*^*tm1Bhk*^/J, strain#021302) mice were obtained from the Jackson Laboratory (Bar Harbor, ME; USA) or the Central Laboratory for Experimental Animals at the Medical University of Warsaw. Prior to bone marrow collection, the mice were housed in the animal facility with a 12-h light/12-h dark cycle (lights on from 7:00 AM to 7:00 PM), with constant access to water and standard rodent food. All studies were conducted in accordance with the European act on the protection of animals used for scientific or educational purposes, as well as the guidelines set forth by the Animal Care and Use Committee of the Warsaw Medical University (Warsaw, Poland).

### Lineage-Negative Cells Depletion

Lin^−^ cells were purified from WT and Nlrp3-KO mice using a Direct Lineage Cell Depletion Kit (Miltenyi Biotec, Bergisch Gladbach, Germany). Briefly, the cells were incubated for 10 min at 4 °C with MicroBeads conjugated to monoclonal antibodies against CD5, CD11b, CD45R (B220), Anti-Gr-1 (Ly-6G/C), and Ter-119 (Direct Lineage Cell Depletion Kit, Miltenyi Biotec, Bergisch Gladbach, Germany). After incubation, the cells were washed with 3 ml of cold phosphate-buffered saline (PBS) and centrifuged at 1800 rpm for 10 min at 4 °C. The cell pellet was resuspended in 1 ml of cold PBS, and the suspension was processed through an autoMACS™ Separator equipped with an autoMACS column (Miltenyi Biotec, Bergisch Gladbach, Germany) to isolate and collect the purified Lin^-^ cell population [17, 18].

### Mitochondria Functionality Measurement—Oxygen Consumption Rate (OCR)

Bone marrow stem cells were isolated from WT and Nlrp3-KO mice. Lin^−^ cells were purified using an autoMACS column, as previously described. The cells were treated with 10 µM H_2_O_2_ for 15 min and then washed with phenol-red-free RPMI media. The oxygen consumption rate was measured at 37 °C using a Seahorse XF HS MINI (Agilent Technologies, Santa Clara, CA, United States) with the Cell Mito Stress Test Kit, following the manufacturer's protocol and as previously described [17, 18]. Briefly, 400,000 cells per well in 180 μl of phenol-red-free RPMI media were added to a Seahorse XFp PDL miniplate and incubated at 37 °C without CO_2_ for 1 h. OCR measurements were taken from a 90% confluent monolayer culture. A protocol was followed to assess mitochondrial function indices, involving sequential injections of oligomycin, FCCP, and a mixture of rotenone and antimycin A through the ports of Seahorse Flux Pak cartridges. The final concentrations of these compounds were 1.5 μM, 2 μM, and 0.5 μM, respectively, which enabled the determination of basal, maximal respiratory, spare respiratory capacity, and ATP production [17, 18].

### Glycolytic Rate Measurements

Bone marrow stem cells were isolated from WT and Nlrp3-KO mice. Lin^−^ cells were subsequently purified using an autoMACS column, as described previously. The cells were then treated with 10 µM H_2_O_2_ for 15 min and washed with phenol-red-free RPMI media. The glycolytic rate was measured at 37 °C using a Seahorse XF HS MINI (Agilent Technologies, Santa Clara, CA, United States) with the Glycolytic Rate Assay Kit, following the manufacturer's protocol. Briefly, 400,000 cells per well in 180 μl of phenol-red-free RPMI media were added to a Seahorse XFp PDL miniplate and incubated at 37 °C without CO_2_ for 1 h. Glycolytic proton efflux rate (glycoPER, pmol/min) measurements were taken from a 90% confluent monolayer culture. A protocol was followed to assess glycolytic rate indices, involving sequential injections of a mixture of rotenone and antimycin A (Rot/AA), followed by 2-deoxy-*D*-glucose (2-DG) through the ports of Seahorse Flux Pak cartridges. The final concentrations of these compounds were 0.5 μM and 50 mM, respectively, enabling the determination of basal and compensatory glycolysis [17, 18].

### Reactive Oxygen Species Level Measurements

The CM-H_2_DCFDA (chloromethyl derivative of 2',7'-dichlorodihydrofluorescein diacetate) (Thermo Fisher Scientific, Cat. No. C6827) was used to measure ROS levels, following the standard procedure. Briefly, Lin^−^ cells (10 000 per 100 µl media) were pre-treated with eATP (1 nM and 10 µM, Merck, Darmstadt, Germany), C3a (1 nM and 10 nM, Bio-Techne, MN, USA), and C5a (1 nM and 10 nM, Bio-Techne, MN, USA) for 16 h. The cells were then centrifuged at 1800 rpm for 10 min, and the resulting cell pellets were resuspended in 1 ml of phenol-red free RPMI medium with CM-H_2_DCFDA (final concentration 5 µM) and incubated at 37 °C for 30 min. Subsequently, the cells were centrifuged (1800 rpm) for 10 min and suspended in 200 µl of phenol-red free RPMI medium. The fluorescence of cells was recorded at λ_ex_ = 488 nm and λ_em_ = 523 nm using SpectraMax iD3 Multi-Mode Microplate Readers (Molecular Devices, CA, USA). ROS levels were quantified using CM-DCF fluorescence intensity and expressed as a percentage of the control (non-treated cells) [].

### H_2_O_2_ Level Measurements

The H_2_O_2_ level in Lin^−^ cells isolated from WT and Nlrp3-KO BM was measured using the ROS-Glo™ H_2_O_2_ Assay (Promega, Madison, WI, USA, Catalog No. G8820) following the manufacturer’s instructions. Control cells (untreated) and cells treated with eATP (1 nM and 10 µM), C5a (1 nM and 10 nM), and C3a (1 nM and 10 nM) were incubated for 16 h at 37 °C in a humidified environment containing 5% CO_2_. After incubation, the cells were harvested, and 20,000 cells were plated per well in a 96-well plate. Then, 20 µl of H_2_O_2_ Substrate Solution was added to each well, and the cells were incubated at 37 °C for 3 h. Subsequently, 100 µl of ROS-Glo™ Detection Solution was added, followed by a 20-min incubation at room temperature. Luminescence intensity was measured using a SpectraMax iD3 Multi-Mode Microplate Reader (Molecular Devices, CA, USA) at 30, 45, and 60 min post-incubation [17, 18].

### Real-Time Analysis of Expression of mRNA for Electron Transport Chain Complexes (ETCs)

Bone marrow Lin^−^ stem cells were isolated from WT and Nlrp3-KO mice. The cells (40,000) were transferred into 200 μl of RLT lysis buffer (Qiagen, Hilden, Germany) and either frozen at − 80 °C or directly subjected to RNA isolation using a RNeasy Micro Kit (Qiagen, Hilden, Germany) following the manufacturer’s protocol. Reverse transcription was performed using the iScript cDNA Synthesis Kit (BioRad, CA, USA), and gene expression was analyzed with the iTaq Universal SYBR Green Supermix (BioRad) along with the primers listed in Table 1 on a CFX Connect real-time cycler (BioRad). The expression of a specific gene in each sample was normalized to the expression of the GAPDH housekeeping gene [17, 18].

**Table 1:** Primers to detect mRNA for electron transport chain complexes

Name	Gene	Primer	Sequence 5’−3’
NADH dehydrogenase 2	**ND2**	mNd2-F mNd2-R	ACCCACGATCAACTGAAGCA AGTACGATGGCCAGGAGGAT
Succinate dehydrogenase (subunit A)	**SDHA**	mSDHa-F mSDHa-R	CACACGTCTACCCGGAATTT CACCTGTCCCTTGTAGTTAGTG
Cytochrome bc1 complex	**CYTB**	mCytB-F mCytB-R	GCTGGTGTACCGTGTCTTCA TGGTAGTCGAACACAGCCAC
Cytochrome c oxidase (subunit I)	**COX1**	mCoxI-F mCoxI-R	TACCCACCTCTAGCCGGAAA CTGTGTTATGGCTGGGGGTT
Cytochrome c oxidase (subunit II)	**COX2**	mCoxII-F mCoxII-R	AGACGAAATCAACAACCCCG GGCAGAACGACTCGGTTATCA
ATP synthase (subunit 8)	**ATP8**	mATP8-F mATP8-R	CATCACAAACATTCCCACTGGC TGAGGCAAATAGATTTTCGTTCATT
Glyceraldehyde 3-phosphate dehydrogenase (housekeeping gene)	**GADPH**	GADPH-F GADPH-R	ACGGCCGCATCTTCTTGTGCA CAAGTGGGCCCCGGCCTTCTC

### Total ATP Production

ATP production in Lin^−^ cells from Nlrp3-KO mice BM was measured using the CellTiter-Glo® 2.0 Cell Viability Assay (Promega, Madison, WI, USA; Catalog No. G9241), following the manufacturer’s protocol. Briefly, Lin^−^ cells (40,000 cells per 100 µl of phenol red-free media) after incubation were centrifuged at 1800 rpm for 10 min, resuspended in an appropriate volume of phenol red-free RPMI medium, and plated into a 96-well plate (100 µl cells/well). Next, 100 µl of CellTiter-Glo reagent was added to each well and mixed gently for 3 min to ensure cell membrane lysis and ATP release. The cells were then incubated at room temperature for 10 min. Luminescence intensity, which indicates ATP levels, was measured using a SpectraMax iD3 Multi-Mode Microplate Reader (Molecular Devices, CA, USA) at 10, 20, and 30 min post-incubation [18].

### Cytochrome c Reductase Assay

The activity of cytochrome c reductase was measured using the Cytochrome c Reductase (NADPH) Assay Kit (Sigma, St. Louis, MO, USA; Catalog No. CY0100) according to the manufacturer’s instructions. Freshly harvested Lin^-^ cells (2.5 × 10^5^ cells per well) isolated from WT and Nlrp3-KO BM were seeded in a 96-well plate for the experiment. Each sample was then mixed with 95 µl of the working solution and 10 µl of NADPH solution (0.85 mg/ml). To prevent interference, an inhibitor solution (cytochrome c oxidase) was added to all samples. Absorbance at 550 nm was measured using a SpectraMax iD3 Multi-Mode Microplate Reader (Molecular Devices, CA, USA) between 1 and 5 min post-incubation. To calculate the results, the following formula was used: Units/µL = [(ΔA550/min) × dil × V_total_]/[21.1 × Enzvol], where: ΔA550/min = A_sample − A_blank, dil = dilution factor of the sample, V_total_ = total reaction volume (µL), 21.1 = extinction coefficient for reduced cytochrome c (mM^−1^·cm^−1^), Enzvol = volume of enzyme added to the reaction (mL).

### Statistical Analysis

All results are presented as the mean ± SD from at least three independent experiments. Statistical analyses were performed using GraphPad Prism 9.0 (GraphPad Software Inc., La Jolla, CA, USA). Data were evaluated using multiple unpaired *t*-tests, with significance set at *p* ≤ 0.05. Statistical significance is indicated as follows: ns – not significant, * *p* < 0.05, ** *p* < 0.01, *** *p* < 0.001, **** *p* < 0.0001.

## Results

### Mitochondrial Activity and Response to Oxidative Stress in WT and Nlrp3-KO Cells

Mitochondrial activity was evaluated in cells isolated from wild-type (WT) and Nlrp3 knockout (Nlrp3-KO) mice under steady-state conditions, as well as after exposure to modifiers of mitochondrial function. The oxygen consumption rate (OCR) was measured during steady-state conditions and sequentially after treatment with key mitochondrial inhibitors and stimulators. Initially, cells were treated with the ATP synthase inhibitor oligomycin to assess basic mitochondrial ATP production. This was followed by treatment with the uncoupler carbonyl cyanide-4-(trifluoromethoxy) phenylhydrazone (FCCP) to induce maximal respiratory capacity. Finally, cells were subjected to rotenone and antimycin A, inhibitors of mitochondrial respiratory chain complexes I and III, respectively, to completely inhibit the electron transport chain. This experimental approach enabled the identification of key mitochondrial parameters in Nlrp3-KO cells, as previously described [17, 18].

**Fig. 1:**
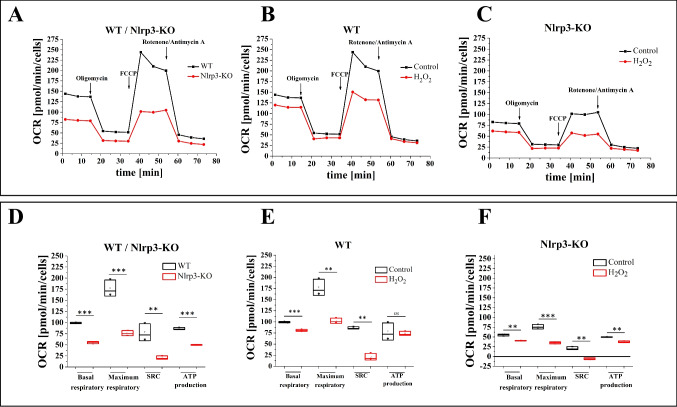
Measurements of oxygen consumption rate (OCR). Mitochondrial activity of Lin.^−^ cells from WT, WT treated with 10 µM H_2_O_2_, Nlrp3-KO, and Nlrp3-KO treated with 10 µM H_2_O_2_ was assessed using a Seahorse XF HS MINI instrument. Panel A) compares OCR in cells isolated from WT and Nlrp3; B) compares WT and WT + 10 μM H_2_O_2;_ and C) compares Nlrp3-KO and Nlrp3-KO + 10 μM H_2_O_2_. Panels D-F) compare basal respiration, maximum respiration, spare respiratory capacity (SRC), and ATP production between cells isolated from WT and Nlrp3-KO mice, as well as those treated with H_2_O_2_. The data represent the mean value ± SD for three independent experiments. Statistical significance is indicated by **p ≤ 0.01, ***p ≤ 0.001

Figure 1A shows that Lin-BM stem cells isolated from Nlrp3-KO mice under steady-state conditions exhibited nearly a twofold decrease in both basal and maximal respiration compared to WT cells. Similarly, Nlrp3-KO cells displayed reduced Spare Respiratory Capacity (SRC) and ATP production relative to WT cells. However, despite these limitations, Nlrp3-KO cells retained some ability to adapt to low or mild oxidative stress, as indicated by SRC values above zero (Fig. 1D).

Reactive oxygen species, including H_2_O_2,_ are produced intracellularly in a NADPH oxidase-2 (Nox2)-dependent manner. They arise from both the activated electron transport chain complexes (ETC) in mitochondria and from Nox2 associated with specific signaling receptors on the cell membrane [11–13]. To further evaluate mitochondrial functionality, we assessed the impact of mild oxidative stress induced by the addition of 10 µM H_2_O_2_ to the cells in vitro (Figs. 1B, 1C and 1E, 1F). Our results indicate that WT cells maintained their ability to adapt to oxidative stress under these conditions, as reflected by SRC values remaining above zero (Fig. 1E). In contrast, Nlrp3-KO cells showed a reduced adaptive capacity, with SRC values slightly below zero (Fig. 1F).

### Glycolytic Adaptations in Wild-Type and Nlrp3 Knockout Cells Under Steady-State and Oxidative Stress Conditions

**Fig. 2:**
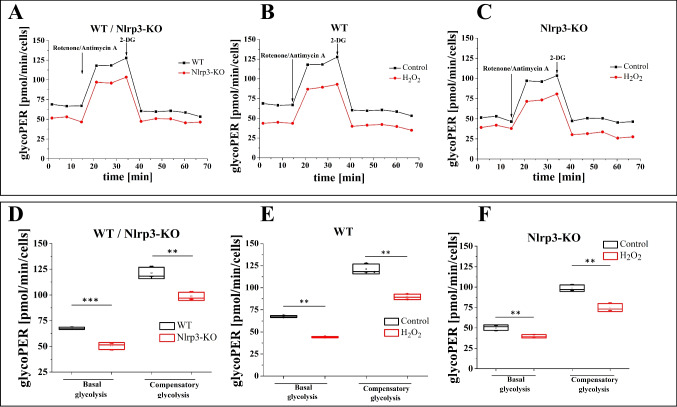
Glycolytic rate measurements. The rate of glycolysis was measured in Lin.^−^ cells from WT, WT treated with 10 µM H_2_O_2_, Nlrp3-KO, and Nlrp3-KO treated with 10 µM H_2_O_2_ using a Seahorse XF HS MINI instrument. Panel A) compares glycoPER in mouse cells isolated from WT and Nlrp3-KO; B) WT and WT + 10 μM H_2_O_2_; and C) Nlrp3-KO and Nlrp3-KO + 10 μM H_2_O_2_. Panels D-F) compare basal and compensatory glycolysis between cells isolated from WT and Nlrp3-KO mice and those treated with H_2_O_2_. The data are presented as mean values ± SD from three independent experiments. Statistical significance is indicated by **p ≤ 0.01, ***p ≤ 0.001

Since impaired oxidative phosphorylation can shift ATP production toward glycolysis [19], we aimed to further investigate the energy metabolism of WT and Nlrp3-KO cells by assessing their glycolytic rates (Fig. 2). We measured the Extracellular Acidification Rate (ECAR) in WT and Nlrp3-KO cells to estimate the glycolytic proton efflux rate (glycoPER) using Seahorse technology.

To evaluate acidification from glycolysis, we initially treated the cells with a combination of rotenone and antimycin A to inhibit mitochondrial respiratory chain complexes I and III, effectively blocking mitochondrial oxygen consumption and proton production from CO_2_. Subsequently, we introduced 2-deoxy-*D*-glucose (2-DG), a well-known inhibitor of glycolysis that competitively binds to glucose hexokinase, the first enzyme in the glycolytic pathway.

Lin-BM stem cells isolated from Nlrp3-KO mice under steady-state conditions demonstrated reduced basal and compensatory glycolysis compared to WT cells (Figs. 2A and 2D). Notably, following exposure to mild oxidative stress (10 µM H_2_O_2_), both WT and Nlrp3-KO cells exhibited decreased glycolytic parameters; however, the reduction was more significant in WT cells than in Nlrp3-KO cells (Figs. 2B, 2C and 2E, 2F).

Considering the results of our Mito Stress test, which indicate that oxidative phosphorylation is likely impaired in Nlrp3-KO cells under oxidative stress conditions while remaining functional in WT cells, this suggests that Nlrp3-KO cells may depend more on glycolysis as an alternative energy pathway. Furthermore, these findings imply that the mitochondria in Nlrp3-KO cells are more susceptible to low concentrations of extracellular H_2_O_2_ than those in WT cells.

### The Effects of C3a, C5a, and eATP on the Production of Various Types of ROS

**Fig. 3:**
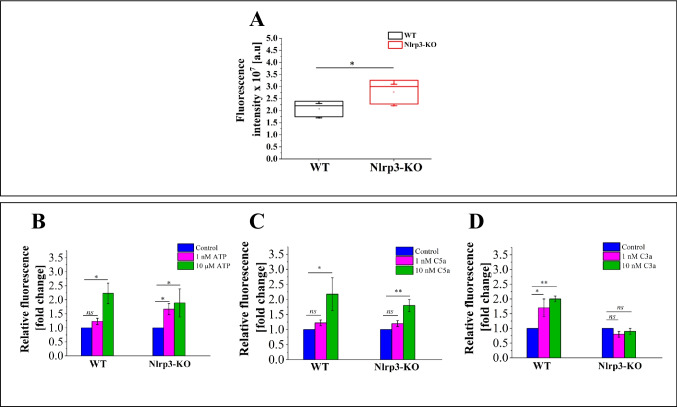
Analysis of all types of ROS levels in bone marrow lineage-negative (Lin.^−^) stem cells isolated from WT and Nlrp3-KO mice. The cells were treated with two concentrations of ATP (1 nM and 10 µM), C5a (1 nM and 10 nM), or C3a (1 nM and 10 nM) for 16 h. Panel A shows relative fluorescence under steady-state conditions. Panel B displays relative fluorescence following treatment with eATP. Panel C presents relative fluorescence after treatment with C5a. Panel D illustrates relative fluorescence after treatment with C3a. Values are expressed as the mean ± SD from at least three independent experiments (*n* = 3). Statistical significance is indicated by *p ≤ 0.05, **p ≤ 0.01

Considering that C3a, C5a, and extracellular ATP (eATP) are well-known stimulators of stem cells that affect HSPC mobilization [20, 21], homing, and engraftment [22, 23], we assessed ROS levels in Lin^−^ cells isolated from WT and Nlrp3-KO mice using the CM-DCF probe. In this assay, we evaluated various types of ROS, including H_2_O_2_, hydroxyl radicals (HO^•^), peroxyl radicals (HOO^•^), and lipid peroxyl radicals (LOO^•^). ROS levels were measured under steady-state conditions (Fig. 3A) and following separate treatment with these stimulators (Figs. 3B–3D).

Our analysis revealed that under steady-state conditions, there was a significant increase in all types of ROS levels in Nlrp3-KO cells compared to WT cells (Fig. 3A). As previously reported [17, 18], WT cells exhibited enhanced ROS production in response to all tested stimulators; however, higher concentrations of eATP (10 µM) and C5a (10 nM) were necessary to trigger a noticeable increase (Figs. 3B and 3C).

In contrast, Nlrp3-KO cells showed increased sensitivity to low concentrations of eATP (1 nM), which were sufficient to induce significant ROS overproduction (Fig. 3B). Like WT cells, Nlrp3-KO cells needed higher concentrations of C5a to trigger an increase in ROS production (Fig. 3C). Surprisingly, Nlrp3-KO cells did not respond to C3a treatment, as ROS levels remained slightly lower than those in untreated controls (Fig. 3D).

### *Evaluation of Targeted ROS (H*_*2*_*O*_*2*_*) Production in WT and Nlrp3-KO Cells Following Stimulation by eATP, C5a, and C3a*

**Fig. 4:**
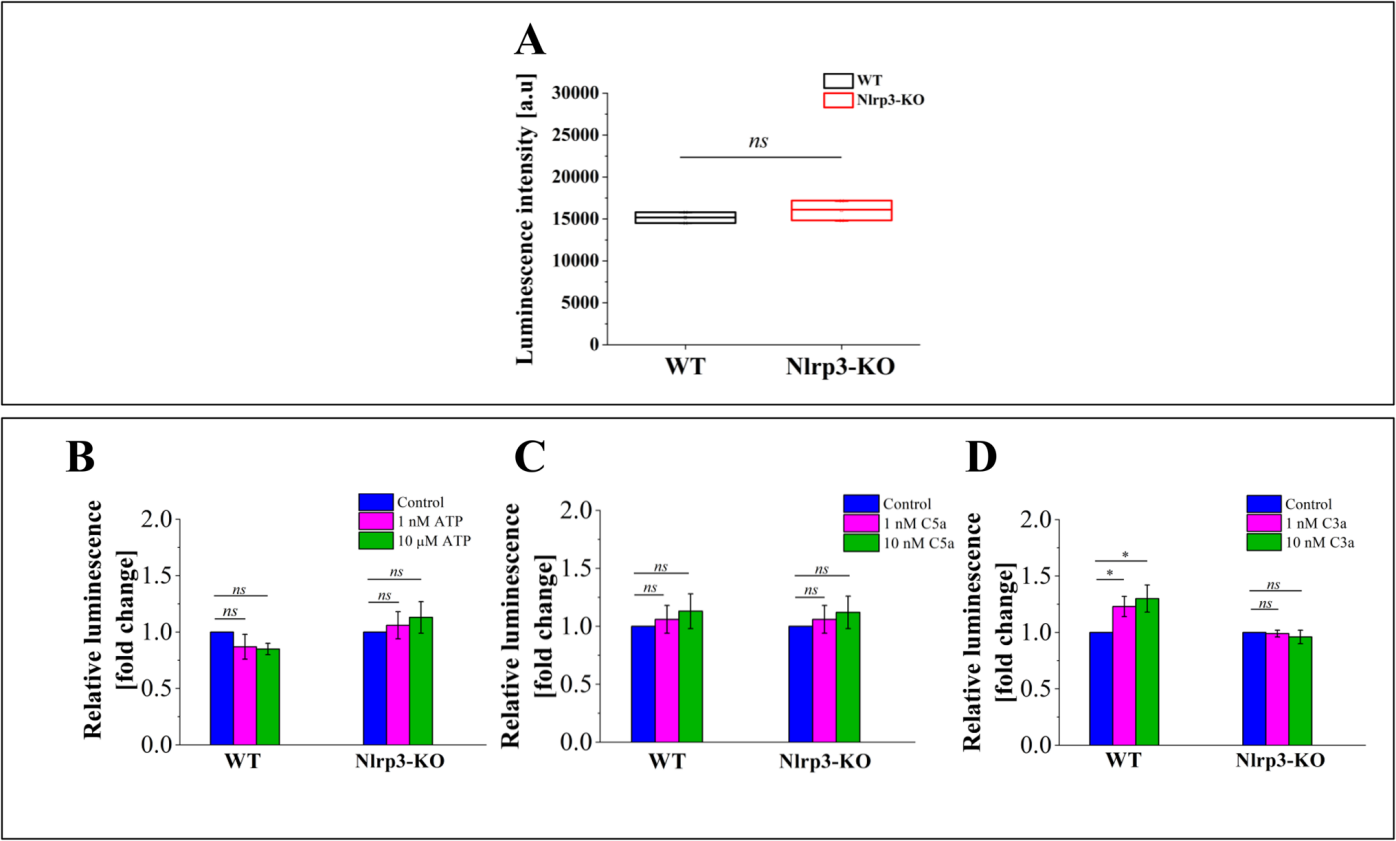
Analysis of the H_2_O_2_ level in bone marrow lineage-negative (Lin.^−^) stem cells isolated from WT and Nlrp3-KO mice. The cells were treated with two concentrations of ATP (1 nM and 10 µM), C5a (1 nM and 10 nM), or C3a (1 nM and 10 nM) for 16 h. **Panel A** shows relative luminescence under steady-state conditions. **Panel B** shows relative luminescence after treatment with eATP. **Panel C** displays relative luminescence after treatment with C5a. **Panel D** presents relative luminescence after treatment with C3a. Values are expressed as the mean ± SD from at least three independent experiments (*n* = 3). Statistical significance is indicated by *p ≤ 0.05

To further investigate the effects of eATP, C5a, and C3a stimulation, we measured H_2_O_2_ levels in WT and Nlrp3-KO cells using the commercially available ROS-Glo™ H_2_O_2_ Assay (Fig. 4). H_2_O_2_ is a well-known representative of ROS and acts as a crucial intracellular signaling molecule involved in various processes, including the regulation of immune cells [19]. Unexpectedly, we observed no significant differences in H_2_O_2_ levels between WT and Nlrp3-KO cells under steady-state conditions (Fig. 4A) or after treatment with eATP, C5a, or C3a (Figs. 4B–4D).

This suggests a contribution from other ROS molecules. Comparing these results to Fig. 3**,** the findings indicate that stimulation with these pro-inflammatory ligands may primarily induce not H_2_O_2_, but rather other types of ROS, such as hydroxyl radicals (HO^•^), peroxyl radicals (HOO^•^), or lipid peroxyl radicals (LOO^•^). Further investigation is needed to confirm this hypothesis, particularly regarding the measurement of SOD responsible for H_2_O_2_ generation.

### Real-Time Analysis of mRNA Expression for Electron Transport Chain Complexes (ETCs) in Wild-Type (WT) and Nlrp3 Knockout (KO) Cells

**Fig. 5:**
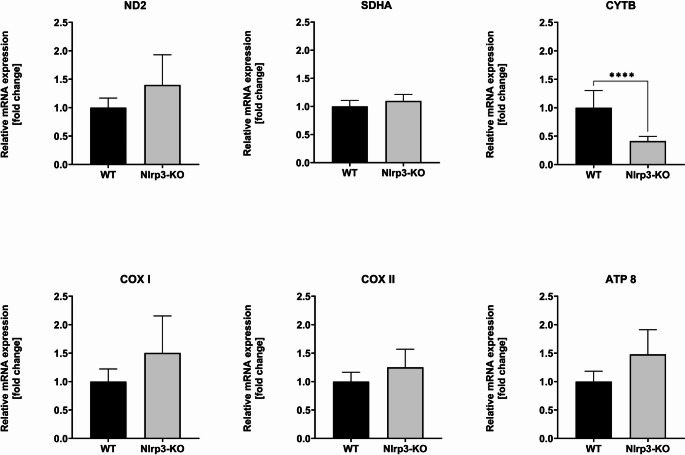
Real-time analysis of mRNA expression for electron transport chain complexes (ETCs) in wild-type (WT) and Nlrp3-KO cells. Relative quantification was performed on samples from WT and Nlrp3-KO mice in triplicate using qPCR to amplify the ND2 (NADH dehydrogenase 2), SDHA (succinate dehydrogenase, subunit A), CYTB (cytochrome bc1 complex), COX1 (cytochrome c oxidase, subunit I), COX2 (cytochrome c oxidase, subunit II), and ATP8 (ATP synthase, subunit 8) genes. These genes are part of the stable component of mtDNA and were normalized against the glyceraldehyde 3-phosphate dehydrogenase gene (GAPDH). Values are expressed as the mean ± SD from at least three independent experiments (*n* = 6) ****p ≤ 0.0001

To clarify the observed defect in mitochondrial function in Nlrp3-KO Lin^−^ BM cells, we employed RQ-PCR to analyze mRNA for the major complexes of the electron transport chain (ETC) (Fig. 5) [24, 25]. We assessed the expression of mRNA for Complex I (NADH dehydrogenase 2), Complex II (succinate dehydrogenase subunit A), Complex III (cytochrome bc1 complex or ubiquinol-cytochrome c reductase), Complex IV (cytochrome c oxidase subunits I and II), and Complex V (ATP synthase subunit 8). We noticed a significant decrease in expression of the mitochondria-encoded cytochrome b gene (CYTB) (Fig. 5), which encodes a crucial component of complex III in the electron transport chain (ETC) [26, 35].

### ETC’s Implications of Decreased CYTB Expression in Nlrp3-KO Lin-BM Cells

**Fig. 6:**
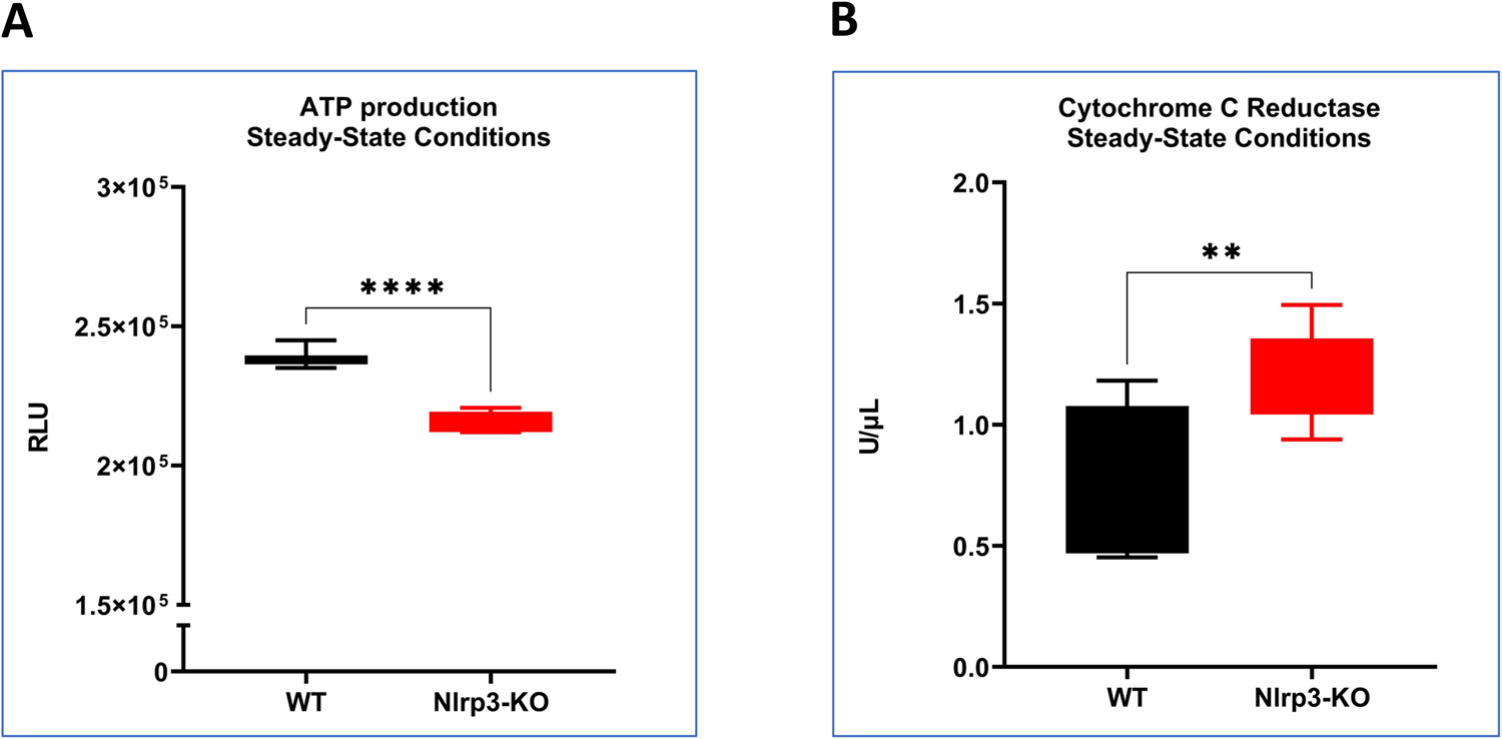
Total ATP production and Cytochrome c Reductase Assay. **Panel A—**Relative luminescence units (RLU) under steady-state conditions. Values are reported as the mean ± standard deviation (SD) from at least three independent experiments (*n* = 3). Statistical analysis was conducted using unpaired t-tests. Statistical significance is indicated by ***p ≤ 0.001. **Panel B—**The activity of cytochrome c reductase was measured using the Cytochrome c Reductase (NADPH) Assay Kit. Values are reported as the mean ± SD from at least three independent experiments (*n* = 3) **p ≤ 0.01)

Complex III of the ETC accepts electrons from Complex II-associated Coenzyme Q and transfers them to Cytochrome C, while simultaneously pumping H^+^ ions derived from FADH_2_ generated in the Krebs cycle [https://www.youtube.com/watch?v=ClBDozXUIWc] into the mitochondrial intermembrane space. This reduction in CYTB expression results in a slight but significant decrease in overall ATP production (Fig. 6A) and is most likely compensated for by an increase in the activity of cytochrome C reductase (Fig. 6B).

## Discussion

The key finding of this paper is evidence showing that Nlrp3 inflammasome expression is essential for maintaining the “tonic activity” of mitochondria in murine BM cells enriched for HSPCs. We observed that the OCR of the mitochondria under steady-state conditions, as well as in response to mild oxidative stress (H_2_O_2_), was lower in Nlrp3-KO cells compared to their WT counterparts. Moreover, the Nlrp3-KO cells exhibited a reduced adaptive capacity to ROS (H_2_O_2_) exposure, with SRC values slightly above zero that became negative following treatment with 10 µM H_2_O_2_. Our evaluation of the Extracellular Acidification Rate (ECAR) regarding the glycolytic proton efflux rate (glycoPER) revealed that Nlrp3-KO cells may depend more on glycolysis as an alternative energy supply pathway. Furthermore, based on the Mito Stress results, we propose that their mitochondria are more susceptible to ROS exposure than those in WT cells.

This decrease in basic and stress-mediated OCR cannot be attributed, as we noticed, to the mitochondrial changes in the expression levels of mRNA for electron transport chain complexes (ETCs). We postulate that this decrease in OCR results from the reduction in Nlrp3 inflammasome-caspase-1-mediated formation of N-gasdermin pores in the cell membrane, which release several alarmins and cytokines to activate corresponding receptors on the cell surface [1, 3, 4, 16, 26]. Some of these alarmins directly stimulate cell metabolism and mitochondrial function (Fig. 6). To support this significant extracellular alarmin, which could be released by N-gasdermin pores—extracellular adenosine triphosphate (eATP)—engaging the P2X7 receptor on the cell surface directly activates the Nlrp3 inflammasome through potassium efflux and indirectly increases OCR by engaging the P2X7 receptor on mitochondria [10]. In fact, we noticed that Nlrp3-KO cells were highly sensitive to low concentrations of eATP (1 nM), which were sufficient to induce ROS overproduction. On the other hand, the baseline level of ROS was higher in Nlrp3-KO cells compared to WT counterparts. This could be explained by the compensatory increase in overall ROS due to a lack of Nlrp3 in the cells. ROS consists of several types of reactive molecules, including H_2_O_2_, hydroxyl radicals (HO^•^), peroxyl radicals (HOO^•^), and lipid peroxyl radicals (LOO^•^) [27, 28], and our data indicate the involvement of all these molecules in the observed phenomena.

We have previously demonstrated that the Nlrp3 inflammasome is expressed in HSPCs and plays a crucial role in regulating the trafficking of these cells, as observed during pharmacological mobilization and homing after transplantation [14]. Furthermore, mice lacking the Nlrp3 inflammasome (Nlrp3-KO) show a reduced number of HSPCs in the bone marrow [14]. Activation of the Nlrp3 inflammasome occurs through ionic fluxes, including K^+^ efflux, Ca2^+^ mobilization, Na^+^ influx, and Cl^−^ efflux, which are noted following the activation of membrane-expressed P2X7 receptors by the major alarmin eATP [10]. However, the primary activators of the Nlrp3 inflammasome are ROS produced by mitochondria that are activated by NADPH oxidase-2 (Nox-2) and after the activation of cell membrane receptors associated with Nox-2 [29, 30]. Key activators of the Nlrp3 inflammasome also include signals from activated extracellular complement cascade cleavage fragments C3a and C5a [30], as well as from intracellular complement (complosome) components C3a and C5a [12, 29, 31]. At low doses, within the beneficial hormetic zone of activation, the effects of the Nlrp3 inflammasome are favorable for cells, influencing cell trafficking and metabolism [4–6]. However, at higher activation levels, the Nlrp3 inflammasome can lead to cell death through “pyroptosis,” which is induced by extensive N-gasdermin pore formation and uncontrolled leakage of cell content into the extracellular space [1, 4].

Our previous data indicates that proper trafficking of HSPCs and their metabolism require maintaining the integrity of the Nox2-ROS-Nlrp3 inflammasome axis [6]. Mice that lack Nox2 and do not generate ROS, similar to Nlrp3-KO mice, show defects in the pharmacological mobilization, homing, and engraftment of HSPCs after transplantation [6]. A similar defect occurs with a deficiency of the crucial alarmin eATP or a lack of its purinergic receptors from the P2X family, including P2X1, P2X4, and P2X7 [32, 33].

**Fig. 7:**
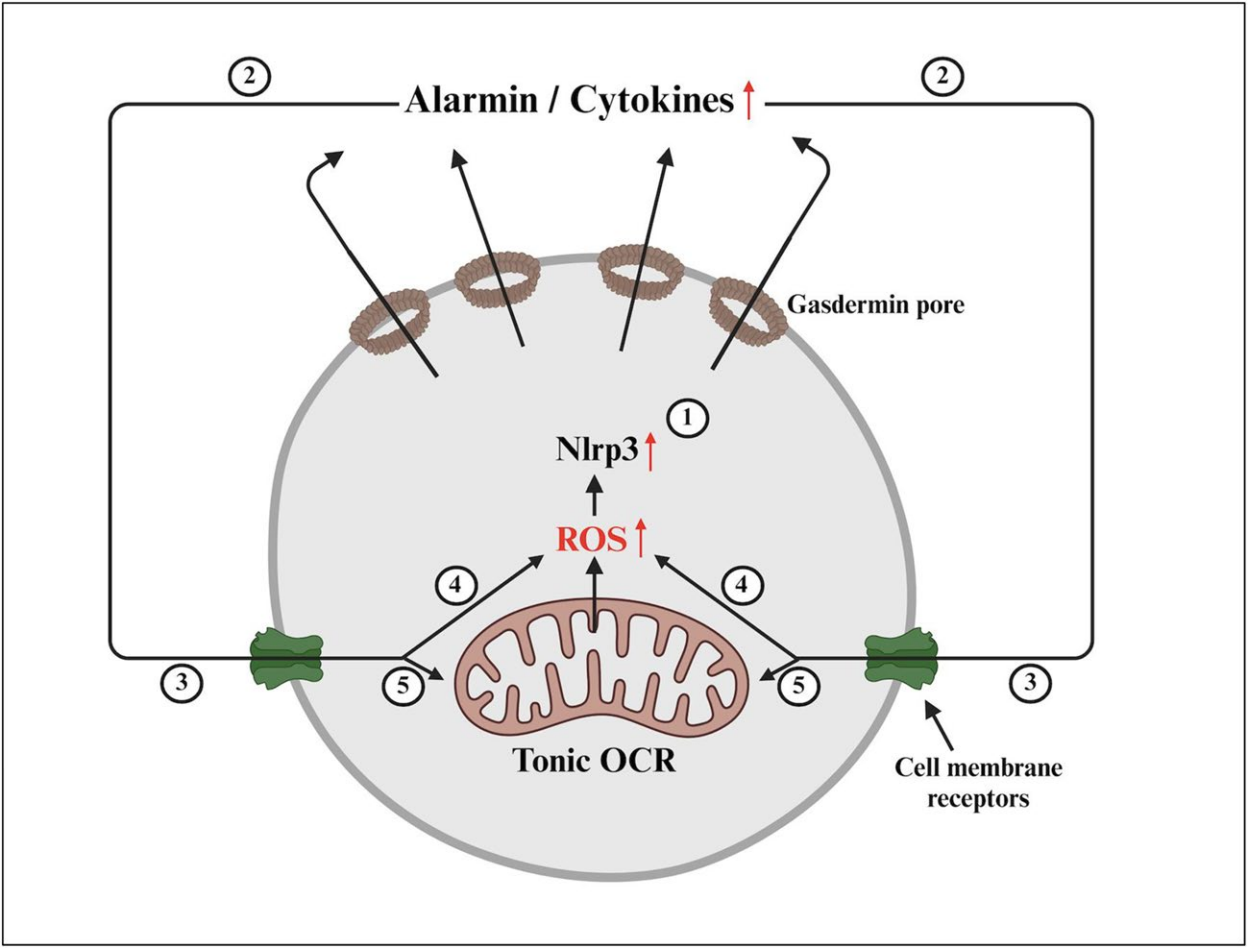
The proposed role of the Nox2-ROS-Nlrp3 inflammasome axis in regulating the biology of HSPCs. Intracellular ROS in HSPCs is produced by Nox-2 expressed in mitochondria and Nox-2 associated with specific cell membrane receptors. These cytosolic ROS activate the Nlrp3 inflammasome, which releases alarmins in a N-gasdermin pores-dependent manner (**1**), including eATP, IL-1β, S100A8/A9, and HMGB-1 (**2**). These alarmins interact with corresponding receptors on the cell surface (**3**), leading to the production of more ROS, which further enhances Nlrp3 inflammasome activation through a positive feedback mechanism. This occurs due to the activity of Nox2 associated with cell membrane receptors (**4**) or directly following the stimulation of mitochondrial Nox-2 (**5**). ROS are important signaling molecules that modify the expression and activity of several enzymes, transcription factors, and structural proteins. These signals within the non-toxic “hormetic zone of activation” enhance the metabolism and migration of HSPCs and maintain a “tonic OCR” in the mitochondria

Understanding the signaling for the activated Nlrp3 inflammasome remains limited. The effector of Nlrp3 activation is caspase-1, which cleaves interleukin-1β and interleukin-18 into active forms that are secreted from the cells and promotes the formation of gasdermin pores to facilitate the release of alarmins and cytokines. If this occurs during a beneficial hormetic phase, it positively impacts the cells [7, 8, 16, 26]. The release of alarmins and pro-inflammatory cytokines, including eATP, increases intracellular levels of ROS through positive feedback (Fig. 7). These ROS are crucial signaling molecules that modify the activity of various enzymes, transcription factors, histones, and structural proteins by oxidizing cysteine and methionine residues in proteins [27, 28]. For example, protein targets for this modification include AKT kinases, NRF2, HIF-1α, FOXOs, AP-1, PTEN, and SIRT1 [27, 28, 34]. Changes in the signaling of these transcription factors may affect the expression of various intracellular and extracellular enzymes.

Our recent data revealed that the activation of the Nox2-ROS-NLRP3 inflammasome axis in HSPC triggers the pentose phosphate pathway, an important intracellular route utilized in anabolic reactions, including the synthesis of structural cholesterol and the lipid components of the cell membrane, particularly those that form membrane lipid rafts (MLRs) [6]. MLRs are essential for optimal signaling of several “raftophilic receptors” on the cell surface. We observed that inhibiting the Nlrp3 inflammasome axis with a small molecular inhibitor MCC950 or the absence of this PRR in HSPCs in KO mice negatively impacts the expression of various enzymes involved in glycolysis, lipidogenesis, and amino acid metabolism [6].

Unexpectedly, no significant differences in intracellular H_2_O_2_ levels were observed between WT and Nlrp3-KO cells under steady-state conditions or after treatment with eATP, C5a, or C3a. These findings imply that stimulation with these compounds may primarily induce other types of ROS, such as hydroxyl radicals (HO^•^), peroxyl radicals (HOO^•^), or lipid peroxyl radicals (LOO^•^), which have a greater ability to reduce the CM-DCF probe to its fluorescent form [34]. This observation aligns more closely with our results presented in Fig. 3. Consequently, we propose that the production of ROS with higher oxidative activity may support our findings regarding changes in the redox status of cells in the Nlrp3-KO background. Nevertheless, further investigation is necessary to support this hypothesis and evaluate the effects of these molecules on the metabolism of HSPCs.

In conclusion, we suggest that the Nlrp3 inflammasome, as a target of ROS signaling, mediates “tonic activation” of OCR through a positive feedback loop involving alarmins released from the cells via gasdermin pores. This process, if it occurs within the beneficial hormetic phase, promotes the trafficking and metabolism of HSPCs [7, 8, 16], and is coordinated by both liver-derived and intracellularly expressed complement [31, 35–37]. It is also important for regulation of cell metabolism because, as already mentioned, inhibition of the Nlrp3 inflammasome in murine SKL cells by MCC950 small molecule inhibitor results in a decrease in the expression of mRNA for several enzymes involved in glycolysis and lipid synthesis [6]. Currently, we are in the process of identifying the signaling consequences of ROS-Nlrp3-gasdermin-released alarmins that regulate the expression of CYTB in BM cells, and one of the potential targets could be transcription factor TFAM.

## Data Availability

Availability of data and materials Detailed data is available upon request.
